# Genetic and Biochemical Approaches for *In Vivo* and *In Vitro* Assessment of Protein Oligomerization: The Ryanodine Receptor Case Study

**DOI:** 10.3791/54271

**Published:** 2016-07-27

**Authors:** Paulina J. Stanczyk, F. Anthony Lai, Spyros Zissimopoulos

**Affiliations:** ^1^Wales Heart Research Institute, Cardiff University School of Medicine, College of Biomedical and Life Sciences

**Keywords:** Molecular Biology, Issue 113, chemical cross-linking, co-immnoprecipitation, mammalian cell transfection, oligomerization, ryanodine receptor, self-association, yeast two-hybrid

## Abstract

Oligomerization is often a structural requirement for proteins to accomplish their specific cellular function. For instance, tetramerization of the ryanodine receptor (RyR) is necessary for the formation of a functional Ca^2+^ release channel pore. Here, we describe detailed protocols for the assessment of protein self-association, including yeast two-hybrid (Y2H), co-immunoprecipitation (co-IP) and chemical cross-linking assays. In the Y2H system, protein self-interaction is detected by β-galactosidase assay in yeast co-expressing GAL4 bait and target fusions of the test protein. Protein self-interaction is further assessed by co-IP using HA- and cMyc-tagged fusions of the test protein co-expressed in mammalian HEK293 cells. The precise stoichiometry of the protein homo-oligomer is examined by cross-linking and SDS-PAGE analysis following expression in HEK293 cells. Using these different but complementary techniques, we have consistently observed the self-association of the RyR N-terminal domain and demonstrated its intrinsic ability to form tetramers. These methods can be applied to protein-protein interaction and homo-oligomerization studies of other mammalian integral membrane proteins.

**Figure:**
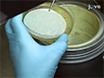


## Introduction

Skeletal and cardiac muscle contraction is triggered by sarcoplasmic reticulum Ca^2+^ release mediated by RyR. There are three mammalian RyR isoforms with the functional channel composed of four identical subunits. Each RyR subunit consists of a large cytoplasmic regulatory N-terminal portion and a small C-terminal part containing the transmembrane domains that form a high-conductance Ca^2+ ^pore. Abnormal intra- and inter-subunit interactions underlie RyR channel dysfunction and result in neuromuscular and cardiac disorders^1^. The identification and characterization of specific domains involved in RyR structure:function relationship is therefore crucial for the understanding of RyR pathophysiology.

Traditional biochemical protein-protein interaction techniques require substantial quantities of purified protein, often produced in bacteria. This is not feasible in the case of the RyR, a very large membrane protein composed of ~5000 amino acids, whereas its recombinant fragments are not easily amenable to bacterial expression and purification. Thus, alternative expression systems involving eukaryotic host cells are required for mammalian integral membrane proteins. We have previously employed Y2H, co-IP and cross-linking assays to collectively demonstrate that N-terminus tetramerization is a structural feature that is conserved across the three mammalian RyR isoforms^2,3^. Importantly, we have found that a single point mutation associated with arrhythmogenic cardiac disease disrupts N-terminus self-association and results in a dysfunctional RyR channel^4^. We have also applied these techniques to oligomerization studies of the RyR cytoplasmic C-terminal tail^5^ as well as the N-terminus of the homologous intracellular Ca^2+^ release channel, the inositol 1,4,5 trisphosphate receptor^2^.

In the Y2H assay, the interaction between two proteins (X and Y) is measured by the reconstitution of a functional transcription factor (GAL4) and the ensuing activation of reporter genes^6-9^. Two different cloning vectors are used to generate fusions of the two tested proteins with the two physically separable, independent domains of GAL4: DNA-Binding Domain (DNA-BD)/protein X fusion (bait) and Activation Domain (AD)/protein Y fusion (target). The Y2H can be used to test whether a protein interacts with itself by generating GAL4 DNA-BD and AD fusions of the same protein. Genetically modified Y2H strains are *GAL4* and *GAL80* deficient (the GAL80 protein is a repressor of GAL4), as well as *TRP1* and *LEU2 *deficient (to provide nutritional selection for bait and prey plasmids, respectively). In the yeast nucleus, the recombinant DNA-BD and AD peptides are brought into close physical proximity to produce a hybrid GAL4 transcription factor only through their fusions' X:Y interaction. This approach enables rapid genetic screening to detect protein-protein interactions through the parallel transcriptional activation of prototrophic (*HIS3* encoding for an enzyme necessary for histidine biosynthesis) and chromogenic (*LacZ* encoding for β-galactosidase (β-Gal)) reporter genes. The main advantage of the Y2H is that it is an *in vivo *assay that detects even weak or transient protein-protein interactions. Moreover, detection involves the simple use of growth selection (in media lacking histidine) or of a colorimetric (β-Gal) assay with no need for purification of the bait and target proteins or the generation of specific antibodies. Additionally, the Y2H can be used to screen a collection of random unknown clones (cDNA library clones fused to GAL4 AD) for novel binding partners of a bait protein, also giving direct access to the cDNA of the library protein.

To extend the Y2H observations, independent biochemical techniques can be employed. Co-IP and cross-linking assays combined with immunoblotting are methods used to detect protein associations in complex sample mixtures, *e.g*., whole cell lysates^10^. Their main advantage is that they report on protein-protein interactions from native tissue, unlike other methods that require the use of recombinant proteins. Recombinant proteins can also be used, typically expressed in a mammalian cell line, where they are likely to have their correct conformation and post-translational modifications, as well as subcellular localization. However, since co-IP and cross-linking are *in vitro* assays making use of homogenized cells, it is necessary to confirm whether the two protein partners are co-localized in the intact cell^11^. We routinely use transfection of mammalian HEK293 cells to transiently express mammalian integral membrane and cytosolic proteins using the calcium phosphate precipitation method^2-4,12-14^, described here in detail. This is an inexpensive way to efficiently deliver the plasmid DNA inside cells but it is dependent on the particular cell line used and cell confluence, as well as the purity of the plasmid DNA^11,15^.

The co-IP assay involves the isolation of the native or recombinant protein of interest from cell lysates under non-denaturing conditions enabling the co-purification of putative interaction partners^10,16^. It requires the use of two independent antibodies, the immunoprecipitating antibody for isolation of protein X, and the immunoblotting antibody for detection of protein partner Y. It can be used to test whether a protein interacts with itself by generating two different fusions with two different epitope tags (*e.g*., HA and cMyc). The immunoprecipitating antibody is bound through its Fc region on Protein-A (or Protein-G, depending on the animal species where the antibody was raised), which is conjugated to agarose (or sepharose) resin. Protein X is precipitated by the antibody:Protein-A resin following incubation with the cell lysate, namely the detergent-soluble fraction of homogenized cells. Protein immuno-complexes are eluted with SDS-containing buffer and subsequently analyzed by SDS-PAGE and immunoblotting using an antibody to detect the presence of protein Y^17^. Co-IP should be carried out with detergent-soluble proteins to avoid excessive non-specific binding. The choice of detergent and its concentration, as well as the number of washes, should be optimized for each X:Y pair^10,16,18^.

Cross-linking is employed to determine the stoichiometry of the oligomeric protein complex. It is based on a chemical reaction to create covalent bonds between adjacent interacting protomers, and therefore, it enables preservation of the protein's oligomeric status during SDS-PAGE separation. There are numerous cross-linking reagents of various lengths and chemistry targeting different reactive groups on proteins, typically primary amines, carboxylic or thiol groups. Here, we describe the use of glutaraldehyde (OHC(CH_2_)_3_CHO), an homo-bi-functional cross-linker with two aldehyde groups on either end that react with free amino groups present in lysine residues^19,20^. Cross-linking is followed in a concentration- or time-dependent manner resulting in adduct formation. Glutaraldehyde reaction is stopped with hydrazine (H_2_NNH_2_) and protein samples are then analyzed by SDS-PAGE and immunoblotting^17^ to evaluate their oligomerization state. We should note that cross-linking does not induce oligomerization but merely creates stable bridges between pre-existing protein complexes. Important considerations when carrying out cross-linking experiments include the choice of cross-linker, its concentration and reaction time^19,20^.

## Protocol

### 1. Yeast Two-hybrid

1. **Yeast Transformation**
  1. Prepare the following media and buffers:
    1. Prepare yeast complete yeast extract-peptone-dextrose (YPD) medium by mixing 20 g/L peptone, 10 g/L yeast extract, 2% w/v glucose (added after autoclaving) and 20 g/L agar (for plates only); sterilize by autoclaving and use fresh on the day of the experiment.
    2. Prepare yeast minimal synthetic defined (SD) medium (lacking leucine and tryptophan to keep selective pressure on both bait and target plasmids) by mixing 6.7 g/L of Yeast Nitrogen Base, 1.6 g/L Dropout supplement lacking leucine and tryptophan, 2% w/v glucose (added after autoclaving) and 20 g/L agar (for plates only); sterilize by autoclaving and use fresh on the day of the experiment.
    3. Prepare 50% w/v PEG (polyethylene glycol) 3350; sterilize through a 0.2 µm filter and store at RT.
    4. Prepare 100 mM Tris/10 mM EDTA (10x TE), adjust the pH to 7.5, sterilize through a 0.2 µm filter and store at RT.
    5. Prepare 1 M lithium acetate (10x LiAc); adjust the pH to 7.5 with CH_3_COOH, sterilize through a 0.2 µm filter and store at RT.
  2. Revive the Y2H strain (*e.g.,* Y190) by streaking a small amount of the frozen glycerol stock onto a YPD plate. Incubate at 30 °C until yeast colonies reach ~2 mm in diameter (usually 3 - 5 days, depending on the yeast strain).
  3. Inoculate 0.5 ml of YPD (in a 1.5 ml tube) with a single, large (2 - 3 mm in diameter) colony. Vortex vigorously for ~2 min to disperse any clumps.
  4. Transfer cell suspension into a 500 ml flask containing 50 ml YPD medium. Incubate at 30 °C for 16 - 18 hr with shaking at 250 rpm for yeast to reach stationary phase.
  5. Transfer 4 - 5 ml of the overnight culture into 200 ml of YPD (in a 1 L conical flask) to produce an optical density at 600 nm (OD_600_, measured using a spectrophotometer) of 0.2 - 0.3 (200 ml will be enough for 20 transformations).
  6. Incubate at 30 °C with shaking at 250 rpm until cells are in mid-log phase, *i.e.*, OD_600_ = 0.5 - 0.6 (usually 2 - 3 hr).
  7. Harvest yeast by centrifugation (in 50 ml tubes) at 1,500 x g for 5 min at RT. Discard the supernatant, resuspend each pellet in 5 ml of sterile H_2_O and pool together.
  8. Re-centrifuge at 1,500 x g for 5 min at RT and discard the supernatant. Resuspend yeast pellet in 1 ml of freshly prepared, sterile 1x LiAc/TE. NOTE: Use competent yeast cells within 1 hr of preparation.
  9. Prepare plasmid samples (in 1.5 ml tubes) by adding 200 ng of plasmid DNA for single transformations, or 0.5 - 1 µg of each plasmid DNA for co-transformations, and 100 µg of herring testes carrier DNA (boiled for 20 min and cooled on ice just prior to use). NOTE: Include a positive control, *e.g*., yeast co-transformed with pVA3 (encoding for GAL4 DNA-BD fusion with p53 protein) and pTD1 (encoding for GAL4 AD fusion with SV40 large T antigen).
  10. Add 100 µl of the freshly prepared, competent yeast suspension (step 1.1.8) and 600 µl of 1x LiAc/PEG solution (8 ml of stock PEG 3350, 1 ml of stock TE, 1 ml of stock LiAc), and vortex for ~30 sec. Incubate at 30 °C for 30 min with shaking at 200 rpm.
  11. Add 80 µl of dimethyl sulfoxide (10% v/v final concentration) and mix well by gentle inversion. Heat shock for 15 min in a 42 °C water bath while mixing every 2 - 3 min.
  12. Chill cell suspension on ice for 2 min and centrifuge at 14,000 x g for 15 sec at RT to recover yeast.
  13. Resuspend cell pellet in 100 µl of 1x TE and plate on appropriate minimal SD medium plates for selective growth (medium lacking leucine and tryptophan to keep selective pressure on both bait and target plasmids).
  14. Incubate plates up-side-down at 30 °C until colonies are ~2 mm in diameter (usually 4 - 5 days). Plates can be stored at 4 °C for 2 - 3 weeks; for longer storage make glycerol stocks and store at -80 °C. NOTE: Verify that bait and target proteins are expressed in yeast by immunoblotting^17^, and that they do not have autonomous reporter gene activation when separately expressed in yeast (by β-Gal assay as described in Section 1.3).
2. **Colony-lift Filter Paper β-Gal Assay**
  1. Prepare the following buffers:
    1. Prepare Z buffer containing 100 mM Na_2_HPO_4_, 40 mM NaH_2_PO_4_, 10 mM KCl, 1 mM MgSO_4_; adjust the pH to 7.4. Sterilize by autoclaving and store at RT.
    2. Prepare X-Gal buffer by dissolving 5-bromo-4-chloro-3-indolyl-β-D-galactopyranoside at 20 mg/ml in N,N-dimethylformamide and store in the dark at -20 °C. Prepare Z buffer/X-Gal solution. Make buffer prior to use by mixing X-Gal at 0.33 mg/ml and β-mercaptoethanol at 0.27% v/v in Z buffer. Use 2.5 ml per sample.
  2. Add 2.5 ml of freshly prepared Z buffer/X-Gal solution in a clean 100 mm plate and place inside a cellulose filter paper.
  3. Place a new filter paper over the surface of the plate with the yeast colonies to be assayed. Gently rub the filter paper onto the plate with forceps and leave for ~5 min for colonies to attach. NOTE: Process the positive control in parallel, *i.e*., yeast co-transformed with pVA3 and pTD1.
  4. Lift the filter paper and submerge it (with forceps) into a pool of liquid nitrogen for 30 sec (liquid nitrogen should be handled with care; always wear thick gloves and goggles). Let the frozen filter paper thaw at RT for ~2 min.
  5. Place the filter paper (colony side up) on top of the pre-soaked filter paper inside the 100 mm plate, and incubate at 30 °C.
  6. Check periodically (every ~30 min) for the appearance of blue colonies. Yeast Y190 co-transformed with the positive control plasmids (pVA3 + pTD1) will turn blue within 60 min (unpublished observations). NOTE: Weak bait:target interactions may take several hours to produce a positive blue signal (avoid prolonged incubation (>8 hr) that may give false positive results). For best results, use freshly co-transformed colonies, *i.e*., grown at 30 °C for 4 - 5 days, ~2 mm in diameter.
3. **Liquid Culture β-Gal Assay**
  1. Prepare the following buffers:
    1. Prepare Z buffer containing 100 mM Na_2_HPO_4_, 40 mM NaH_2_PO_4_, 10 mM KCl, 1 mM MgSO_4_; adjust the pH to 7.4, sterilize by autoclaving and store at RT. 1 M Na_2_CO_3_; store at RT.
    2. Prepare Z buffer/β-mercaptoethanol. Make buffer prior to use by adding β-mercaptoethanol at 0.27% v/v in Z buffer; use 700 µl per sample. Prepare Z buffer/ONPG solution. Make buffer prior to use by mixing ONPG (o-nitrophenyl-β-D-galactopyranoside) at 4 mg/ml and β-mercaptoethanol at 0.27% v/v in Z buffer; use 160 µl per sample.
  2. Use a single colony to inoculate 5 ml of minimal SD medium (lacking leucine and tryptophan to keep selective pressure on both bait and target plasmids) and incubate at 30°C for 16 - 18 hr with shaking at 250 rpm. NOTE: Assay five separate colonies co-transformed with the same bait and target plasmids.
  3. Transfer enough of the overnight culture into 10 ml of fresh SD medium to produce an OD_600_ = 0.2 - 0.3. Incubate at 30 °C with shaking at 250 rpm until the cells are in mid-log phase (OD_600 _= 0.5 - 0.6).
  4. Transfer 0.5 ml of yeast culture into a 1.5 ml tube and centrifuge at 14,000 x g for 2 min at RT. Record the exact OD_600 _when harvesting the cells. Resuspend pellet in 100µl of Z buffer; this will result in a 5-fold concentration factor.
  5. Place tube in liquid nitrogen for ~1 min (liquid nitrogen should be handled with care; always wear thick gloves and goggles) and then in a 37 °C water bath for 3 min to thaw. Repeat this freeze-thaw cycle twice more to ensure cells are broken open.
  6. Set up a blank tube with 100 µl of Z buffer.
  7. Add 700 µl of Z buffer/β-mercaptoethanol and 160 µl of Z buffer/ONPG to the sample and blank tubes; start the timer and place in a 30 °C incubator.
  8. Check periodically for yellow color to develop. Add 400 µl of 1 M Na_2_CO_3_ to stop color development and record elapsed time in minutes. Yeast Y190 co-transformed with the positive control plasmids (pVA3 + pTD1) will turn yellow within 60 min.. NOTE: Weak bait:target interactions may take several hours to produce a positive yellow signal For best results, use freshly co-transformed colonies, *i.e*., grown at 30 °C for 4 - 5 days, ~2 mm in diameter.
  9. Centrifuge at 14,000 x g for 5 min at RT to pellet cell debris and transfer the supernatant into a clean cuvette.
  10. Using a spectrophotometer, measure the absorbance at 420 nm (A_420_) of the samples relative to the blank (values should be between 0.02 - 1.0).
  11. Calculate β-galactosidase units, with 1 unit defined as the amount which hydrolyses 1 µmol of ONPG to o-nitrophenol and D-galactose per min per cell, according to the following formula: 
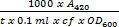
 = β-galactosidase units Where: t: elapsed time of incubation (in minutes); cf: the concentration factor from step 1.4.8, *i.e*., cf = 5; OD_600_: when cells were harvested.

### 2. Protein Expression in a Mammalian Cell Line

1. **Mammalian Cell Transfection**
  1. Prepare the following media and buffers:
    1. Prepare growth medium by mixing DMEM with 4.5 g/L glucose, 10% v/v fetal bovine serum and 2 mM L-glutamine; sterilize through a 0.2 µm filter and store at 4 °C.
    2. Prepare 2x Hepes-buffered saline (2x HBS) by mixing 280 mM NaCl, 10 mM KCl, 1.5 mM Na_2_HPO_4_, 12 mM glucose, 50 mM Hepes; adjust the pH to 7.05, sterilize through a 0.2 µm filter and store at -20 °C. Prepare 2.5 M CaCl_2._ Sterilize through a 0.2 µm filter and store at -20 °C.
  2. One day before transfection, seed 1 - 2 x 10^6^ HEK293 cells in a 100 mm Petri dish in order to be 60-70% confluent the following day. Culture for 16 - 18 hr at 37 °C in a humidified incubator with 5% CO_2_.
  3. On the day of transfection, remove the old medium and feed cells with 10 ml of fresh growth medium. NOTE: Antibiotics are omitted from the culture medium during transfection because they may increase cell death.
  4. Dilute 24 µg of plasmid DNA (for co-transfections, an equal molar ratio of the two plasmids) and 60 µl of 2.5 M CaCl_2_ in 600 µl total volume (made with sterile deionized H_2_O) inside a 1.5 ml tube; vortex to mix. NOTE: For highest transfection efficiency, plasmid DNA should be of the highest purity, *i.e*., have a Abs_260_/Abs_280_ ratio = ~1.8.
  5. Add the plasmid DNA/calcium solution drop wise into a 50 ml tube containing 600 µl of 2x HBS while constantly and vigorously mixing by vortexing. Incubate for 20 min at RT to allow formation of calcium phosphate/plasmid DNA complexes.
  6. After the 20 min incubation, vortex briefly and add the solution drop wise onto the cells to cover the whole surface area of the 100 mm Petri dish.
  7. Incubate the cells at 37 °C in 5% CO_2_. After ~6 hr change the growth medium and place back in the incubator.
  8. Harvest the cells 24 hr post-transfection by centrifugation at 1,000 x g for 3 min at RT and discard the supernatant. Cell pellets can be stored at -80 °C until needed. NOTE: Expression usually peaks 24 - 72 hr post-transfection.
2. **Cell Homogenization**
  1. Prepare the following buffers:
    1. Prepare Co-IP homogenization buffer by mixing 150 mM NaCl, 20 mM Tris; adjust the pH to 7.4 and store at 4 °C.
    2. Prepare Cross-linking homogenization buffer by mixing 5 mM Hepes, 0.3 M sucrose; adjust the pH to 7.4 and store at 4 °C (filter before use to remove any particulates). Supplement with protease inhibitors prior to use.
  2. Add 250 µl of glass beads (425 - 600 microns) inside a 1.5 ml tube and wash with 500 µl of homogenization buffer. Pellet glass beads by a brief centrifugation pulse (1,000 x g for 5 sec) and remove the supernatant. Repeat this wash step once more.
  3. Resuspend cell pellet (from step 2.1.8) in 500 µl of ice-cold homogenization buffer and transfer the cell suspension into the glass beads-containing tube.
  4. Homogenize cells on ice by 20 passages through a fine needle (23 G, 0.6 x 30 mm) attached to a 1 ml syringe. With the tube cap closed, pierce through it with the needle and disperse cell suspension vigorously through the glass beads.
  5. Centrifuge at 1,500 x g for 10 min at 4 °C to remove nuclei and unbroken cells and discard the pellet. Save the supernatant representing the post-nuclear fraction and proceed directly to co-IP or cross-linking as appropriate.

### 3. *In vitro* Biochemical Methods

1. **Co-immunoprecipitation**
  1. Prepare the following buffers:
    1. Prepare Co-IP homogenization buffer as described in section 2.2.1.1. Prepare IP buffer by mixing 20 mM Tris, 150 mM NaCl, 0.5% (w/v) CHAPS, 2 mM dithiothreitol (optional; dithiothreitol can be included to reduce protein aggregates that may have formed due to air oxidation); adjust the pH to 7.4 and store at 4 °C.
    2. Prepare 2% w/v CHAPS in co-IP homogenization buffer; store at 4 °C.
    3. Prepare Protein-loading buffer by mixing 60 mM Tris, 2% w/v SDS, 10% v/v glycerol, 5 mM EDTA, 0.01% w/v bromophenol blue, 2% v/v β-mercaptoethanol (optional); adjust the pH to 6.8 and store at RT.
  2. Homogenize the cells from a confluent 100 mm Petri dish (~8 x 10^6^ cells if HEK293, counted with the use of a hemocytometer) as described in Section 2.2.
  3. Solubilize the post-nuclear sub-cellular fraction with 0.5% CHAPS (using the 2% stock) and incubation for ≥2 hr at 4 °C with constant mixing. Centrifuge at 20,000 x g for 10 min at 4 °C to pellet the insoluble material and discard the pellet. Save the supernatant termed cell lysate. NOTE: The inclusion of a detergent and removal of the insoluble material is absolutely necessary to minimize non-specific binding. Intermediate detergents, *e.g*., CHAPS or Triton X-100, at a concentration of 0.2 - 1%, are the most commonly used.
  4. Prepare two separate 1.5 ml tubes and add ~20 µl (depending on IgG binding capacity) of 6 mg/ml Protein-A or Protein-G agarose (or sepharose) beads. Wash with 200 µl of IP buffer. NOTE: Choose the appropriate Ig-binding resin depending on the animal species used to raise the immunoprecipitating antibody. Protein-G binds a broader range of Ig subtypes compared to Protein-A.
  5. Recover beads by centrifugation at 1,500 x g for 2 min at 4 °C. Repeat wash once more with IP buffer, then resuspend beads in 200 µl of IP buffer.
  6. Add 1 µg (5 ng/µl) of the immunoprecipitating antibody or non-immune IgG (to serve as negative control) in the two separate Protein-A/G containing tubes. Incubate for ≥2 hr at 4 °C with constant mixing. NOTE: Always process a negative control with the use of non-immune IgG raised in the same animal species as the immunoprecipitating antibody.
  7. Recover beads by centrifugation at 1,500 x g for 2 min at 4 °C and carefully discard the supernatant by pipetting.
  8. Transfer 200 µl of cell lysate (from step 3.1.3) into each of the two tubes with antibody and Protein-A/G beads. Incubate for ≥2 hr at 4 °C with constant mixing to allow for antigen-antibody binding.
  9. Recover beads and wash with 200 µl of IP buffer; incubate for 10 min at 4 °C with mixing. Recover beads and wash twice more (avoid multiple washes which will reduce both the specific and non-specific binding). Carefully discard the supernatant by pipetting.
  10. Add 30 µl of protein-loading buffer to elute immunoprecipitated proteins. Centrifuge at 1,500 x g for 2 min at 4 °C, discard the beads and save the supernatant containing the eluted co-IP sample.
  11. To verify successful protein X precipitation, load a small amount (1/10^th^, 3 µl) of the co-IP sample on an SDS-PAGE gel to be analyzed by immunoblotting^17^ with antibody specific for protein X. Include an aliquot of the cell lysate to verify protein X expression in your sample.
  12. To test for the presence of co-precipitated protein Y, load the rest (9/10^th^, 27 µl) of the co-IP sample on a separate SDS-PAGE gel to be analyzed by immunoblotting^17^ with antibody specific for protein Y. Include an aliquot of the cell lysate to verify protein Y expression in your sample.
2. **Chemical Cross-linking**
  1. Prepare the following buffers:
    1. Cross-linking homogenization buffer as described in section 2.2.1.1.
    2. Prepare 5x protein-loading buffer by mixing 300 mM Tris, 10% w/v SDS, 50% v/v glycerol, 25 mM EDTA, 0.05% w/v bromophenol blue, 10% v/v β-mercaptoethanol (optional); adjust the pH to 6.8 and store at RT; warm at 50 °C before use.
  2. Homogenize the cells from a confluent 100 mm Petri dish (~8 x 10^6^ cells if HEK293, counted with the use of a hemocytometer) as described in Section 2.2. NOTE: Sucrose (at 0.3M) is used to create an iso-osmotic buffer. Salt, *e.g.*, 120 - 150 mM NaCl or KCl can be used instead, depending on the oligomeric protein complex of interest.
  3. Centrifuge the post-nuclear sub-cellular fraction at 20,000 x g for 10 min at 4 °C to pellet protein aggregates and save the supernatant. Take eight aliquots of 20 µl each (typically ~20 µg of protein) into separate 0.5 ml tubes.
  4. Add 0.0025% v/v glutaraldehyde to all samples and start the timer. Let each of the eight samples react with glutaraldehyde at RT for: 0, 2, 5, 10, 15, 20, 30, 60 min. NOTE: Glutaraldehyde has two aldehyde groups that react with free amines. Avoid using pH buffers or other substances with primary amino groups because they will quench the glutaraldehyde reaction.
  5. Stop glutaraldehyde reaction at the specified time with 2% v/v hydrazine and add 5 µl of 5x protein-loading buffer to denature proteins.
  6. Proceed to SDS-PAGE and immunoblotting^17^.

## Representative Results

In the Y2H system, the bait:prey interaction is initially tested by yeast growth selection in medium lacking (tryptophan, leucine and) histidine and subsequently assessed by β-Gal enzymatic activity assays (**Figure 1**). Yeast cultured in medium lacking histidine has slow growth rate that depends on the strength of the bait:prey protein interaction. The β-Gal assay is carried out in yeast (cultured in medium lacking only tryptophan and leucine) either growing on solid support (agar plates) or in liquid culture, with the latter yielding quantitative results. We have successfully used the Y2H to identify domain-domain interactions within the RyR2 as well as novel protein partners^2-4,12,21,22^. For example, we screened a series of overlapping constructs spanning the entire length of the RyR2 peptide sequence for interaction with an N-terminal fragment (AD4L: RyR2 residues 1 - 906 fused with GAL4 AD)^3^. Colony-lift filter paper β-Gal assays produced vivid blue-coloured yeast colonies only for the BT4L:AD4L pair (**Figure 2A**), showing that AD4L interacts with itself, namely the BT4L construct (RyR2 residues 1 - 906 fused with GAL4 DNA-BD). Pale blue colonies were detected for the BT8:AD4L pair suggesting a secondary weaker association with the extreme C-terminal domain (BT8), whereas yeast co-transformed with any other construct remained white and therefore negative for bait:prey protein interaction. Quantitative results, obtained by liquid β-Gal assays (**Figure 2B**), indicated robust BT4L:AD4L interaction equivalent in strength to the known association between the p53 protein (pVA3) and SV40 large T antigen (pTD1), whereas the BT8:AD4L interaction was considerably weaker (<10% compared to control).

We routinely carry out co-IP experiments (**Figure 3**) following transient expression in a mammalian cell line (HEK293), as an independent biochemical assay to reinforce the Y2H findings^2-4,12-14,21-24^. To verify RyR2 N-terminus self-interaction, two separate plasmids encoding for RyR2 residues 1 - 906 tagged with either the cMyc or HA peptide epitope (BT4L and AD4L, respectively), were co-transfected in HEK293 cells using the calcium phosphate precipitation method^3^. The post-nuclear fraction of homogenized cells was solubilized with the detergent CHAPS, and the insoluble material was removed by centrifugation to produce the cell lysate. The cell lysate, treated with the reducing agent dithiothreitol, was then incubated with Ab^HA^ and Protein-A sepharose beads to immunoprecipitate HA-tagged AD4L. Co-IP samples, eluted with SDS-containing buffer, were loaded on two separate SDS-PAGE gels (1/10^th^ and 9/10^th^ split) and analyzed by immunoblotting with Ab^HA^ and Ab^cMyc^, respectively. Successful direct IP of AD4L (~100 kDa) by Ab^HA^ was verified by immunoblotting, but not in the negative control using non-immune rabbit IgG (**Figure 4A**). Importantly, cMyc-tagged BT4L (~100 kDa) was recovered only in the Ab^HA^ IP, and not in the negative control in the absence of immunoprecipitated AD4L (**Figure 4B**).

Y2H and co-IP assays provided consistent evidence for RyR2 N-terminus self-interaction, but they did not inform on the oligomerization status of the N-terminal domain, namely whether it forms only dimers or higher complexes. It should be noted that complexes will dissociate and only the comprising subunits will be detected by SDS-PAGE because of SDS- and heat-induced protein denaturation abolishing protein-protein interactions. To overcome this, we use cross-linking (**Figure 5**) that chemically and stably conjoins pre-existing protein oligomers, whose molecular mass can then be examined by SDS-PAGE size separation^2-5^. For example, HEK293 cell homogenate expressing cMyc-BT4L, treated with the reducing agent dithiothreitol, was reacted with glutaraldehyde and analyzed by SDS-PAGE and immunoblotting using Ab^cMyc^ (**Figure 6**). In addition to the monomer (~100 kDa), a high molecular mass protein band of ~400 kDa was detected in a time dependent manner, indicating RyR2 N-terminus tetramer formation^3^. Notably, tetramer was the predominant oligomeric species, with minimal dimer and trimer bands observed. To determine its apparent molecular mass, the BT4L oligomer was separated through 4 - 15% gradient SDS-PAGE gels^3^. We produced the molecular mass/gel retardation standard curve using protein standards with a range of 30 - 460 kDa, and we calculated the oligomer to be 358 kDa 15 (n = 4). This apparent molecular mass is consistent with a BT4L tetramer arranged in a closed circular fashion rather than in linear form, as expected from the arrangement of the four subunits within the native RyR2 channel.


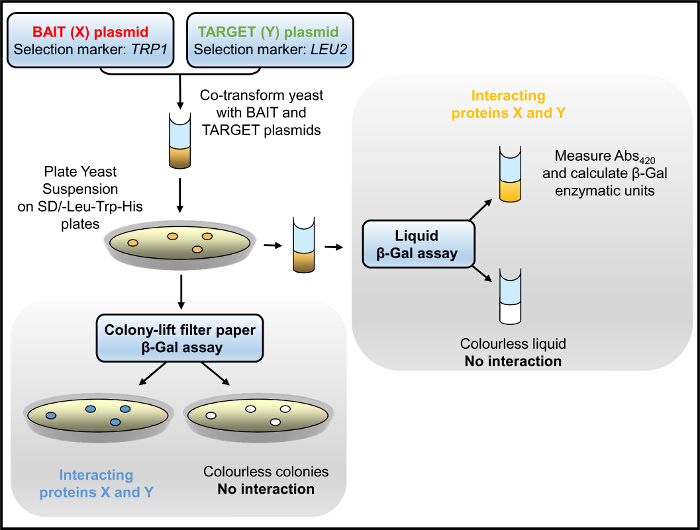
**Figure 1.Y2H Flowchart. **Yeast, co-transformed with bait and target plasmids, is selected for growth in medium lacking histidine and/or assayed for β-Gal enzymatic activity. Please click here to view a larger version of this figure.


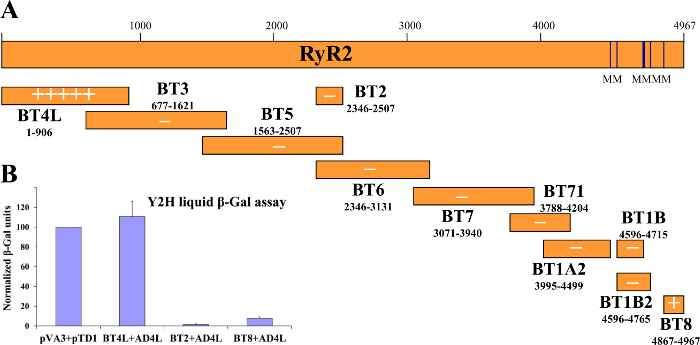
**Figure 2.Y2H Suggests RyR2 N-terminus Domain Self-interaction. (A)** Schematic depicting the (bait) series of human RyR2 overlapping protein fragments tested in the Y2H system for interaction with the RyR2 N-terminal AD4L (prey) construct. Qualitative results obtained by colony-lift filter paper β-Gal assays are indicated in "+" multiples or "-" for negative interaction. **(B)** Quantitative liquid β-Gal assays normalized against the positive control (pVA3 encodes for GAL4 DNA-BD fusion with p53 protein; pTD1 encodes for GAL4 AD fusion with SV40 large T antigen). Modified from^3^. Please click here to view a larger version of this figure.


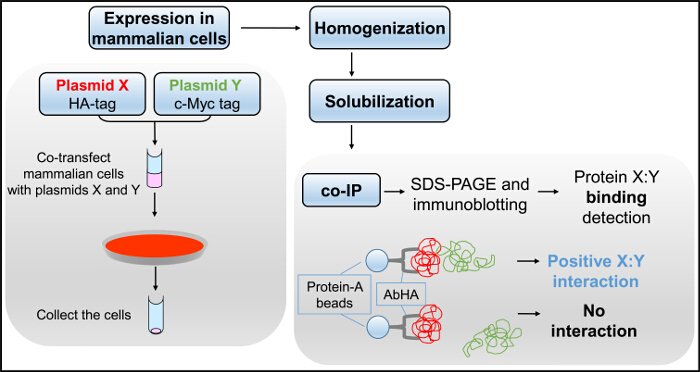
**Figure 3. Co-IP Flowchart. **Mammalian cells, co-transfected with plasmids X and Y, are homogenized and detergent-solubilized to produce the cell lysate used in the co-IP assay, followed by SDS-PAGE and immunoblotting. Please click here to view a larger version of this figure.


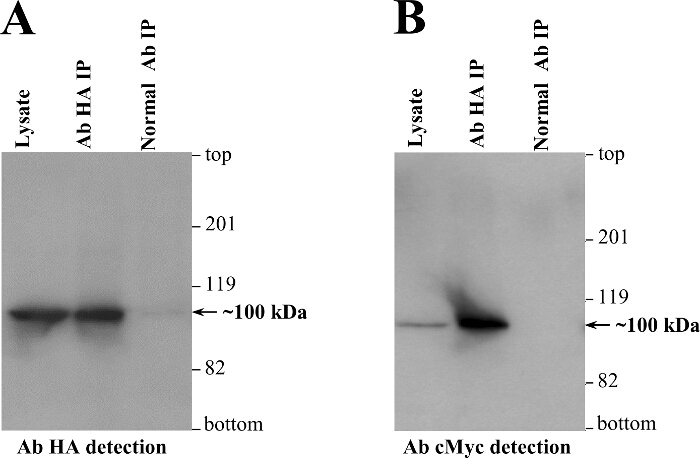
**Figure 4.Co-IP Indicates RyR2 N-terminus Domain Self-interaction in Mammalian Cells. **HEK293 cells were co-transfected for transient co-expression of cMyc-tagged (BT4L) and HA-tagged (AD4L) RyR2 N-terminus domain (residues 1 - 906). AD4L was immunoprecipitated with Ab^HA^ from CHAPS-solubilized and dithiothreitol-treated HEK293 lysate, whereas as negative control, co-IP assays were carried out with non-immune rabbit IgG. Immunoprecipitated proteins were heated at 85 °C for 5 min and resolved at 20 mA for 3 hr through separate 6% SDS-PAGE gels loaded with 1/10^th^ or 9/10^th^ of IP samples. Following protein transfer at 80 V for 2 hr onto polyvinylidene difluoride membrane, immunoblotting analysis was carried out using (1:1,000 dilution) Ab^HA^**(A)** or Ab^cMyc ^**(B)**, respectively, followed by horseradish peroxidase-conjugated anti-mouse IgG (1:10,000 dilution) and enhanced chemiluminescence detection (1 min exposure). An aliquot of cell lysate, 1/50^th^ of the volume processed in IP samples, was also included to serve as molecular mass standard. Modified from^3^. Please click here to view a larger version of this figure.


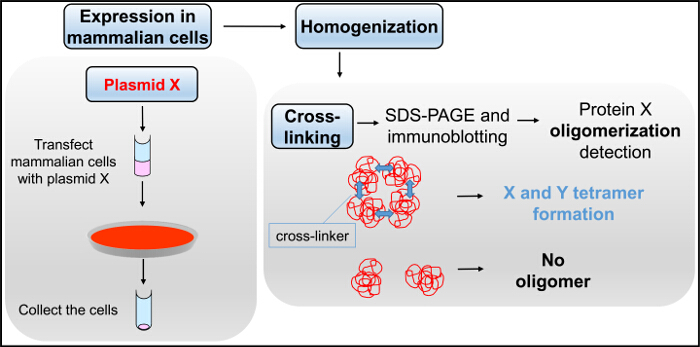
**Figure 5.Cross-linking Flowchart. **Mammalian cells, transfected with plasmid X, are homogenized and subjected to reaction with glutaraldehyde in the cross-linking assay, followed by SDS-PAGE and immunoblotting. Please click here to view a larger version of this figure.


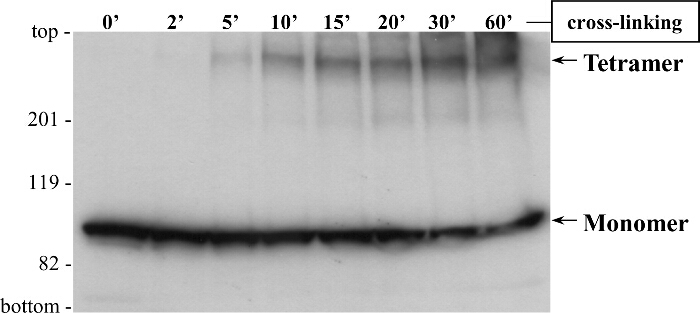
**Figure 6. Cross-linking Indicates RyR2 N-terminus Domain Tetramer Formation. **HEK293 cells were transfected for transient expression of cMyc-tagged (BT4L) RyR2 N-terminus domain (residues 1 - 906). Cell homogenate, treated with the reducing agent dithiothreitol, was incubated with glutaraldehyde for the indicated time points. Samples were heated at 85 °C for 5 min and resolved by SDS-PAGE (6% gel) at 20 mA for 3 hr. Following protein transfer at 80 V for 2 hr onto polyvinylidene difluoride membrane, immunoblotting analysis was carried out using (1:1,000 dilution) Ab^cMyc^, followed by horseradish peroxidase-conjugated anti-mouse IgG (1:10,000 dilution) and enhanced chemiluminescence detection (1 min exposure). Monomeric (M: ~100 kDa) and tetrameric (T) forms are indicated by the arrows. Modified from^3^. Please click here to view a larger version of this figure.

## Discussion

The formation of protein homo-oligomers is a fundamental biological process that regulates the activity of transcription factors, enzymes, ion channels and receptors^25,26^. Importantly, protein oligomerization has also pathological implications including neurodegeneration and arrhythmogenic cardiac disease^4,27^. The methodologies outlined in this article are used to identify domain-domain interactions mediating protein self-association and oligomerization. Below, we point at critical steps within each protocol, and we discuss important considerations, limitations and troubleshooting.

The Y2H system can be employed first to screen for potential interacting protein partners because of its relatively high throughput screening, ease of use and highly reproducible results. Y2H procedures are carried out in a microbiology laboratory with standard (plate or shaker) incubators and room containment facilities. The use of freshly prepared competent cells (step 1.1.8) is critical for high efficiency yeast transformation, whereas for best results in β-Gal assays, freshly grown yeast colonies (up to 5 days old) should be used (step 1.2.3). Systems based on transcription factors other than GAL4 and/or additional/alternative reporter genes are available, and therefore bait and prey plasmids should be matched with the appropriate Y2H strain.

Some strains should be cultured in the presence of 3-amino-1,2,4-triazole, a competitive inhibitor of the HIS3 protein, in order to quench any constitutive expression of the *HIS3* reporter gene^7,8^. Expression of bait and target fusion proteins should be verified by immunoblotting^17^. In case bait/prey fusion proteins are toxic to yeast, lower tolerable protein levels could be achieved by cloning in different vectors where bait/prey expression is driven by a different promoter. Further, it is essential to ensure that bait/prey fusion proteins do not display autonomous reporter gene activity. Autonomous reporter gene activation can be rescued by modifying the construct to remove the responsible region, or by swapping the GAL4 DNA-BD and AD fusions for the two tested proteins. Moreover, transmembrane domains are better omitted from bait/prey constructs because they may induce misfolding or mislocalization of fusion proteins in intracellular membrane compartments. Indeed, the main disadvantage of the Y2H system is that the bait and target proteins are localized in the yeast nucleus away from their physiological subcellular location and potentially lacking specific post-translational modifications, resulting in false-positive or false-negative interactions^6-9^.

Mammalian heterologous expression systems are better suited for the study of mammalian integral membrane proteins in terms of conformation, post-translational modifications and subcellular localization. One of the most widely used cell transfection methods is the calcium phosphate precipitation, mainly because of the minimum equipment and reagents required^11,15^. Alternative methods, namely electroporation, liposomes, cationic lipids and polymers, may yield higher transfection efficiency depending on the cell line and construct used. In general, the primary factors affecting transfection efficiency are plasmid DNA quality and cell health/viability. The best results are achieved when plasmid DNA of the highest purity (260nm/280nm absorbance ratio of ~1.8) and actively dividing cells are used. Cells should therefore be transfected at no more than 60 - 70% confluence (step 2.1.2), because the ability to take up foreign DNA is related to the surface area of the cell exposed to the medium^11^. Additionally, inclusion of antibiotics in the culture medium during transfection (step 2.1.3) is not advised due to increased cell death^15^.

For calcium phosphate precipitation in particular, careful preparation and pH adjustment (to 7.05 precisely) of the 2x HBS solution (step 2.1.1), and proper formation of plasmid DNA/calcium/phosphate complexes by vigorous mixing (step 2.1.5) are critical steps for high efficiency transfection. Typically, protein expression by transient transfection peaks within 24 - 72 hr.

Once cells are harvested, subsequent procedures must be carried out at 4°C to minimize protease activity, and addition of protease inhibitors is recommended. Cell homogenization should be followed by a centrifugation step to remove nuclei because chromosomal DNA may increase solution viscosity and enhance non-specific binding. Thus, homogenization by mechanical means in an iso-osmotic buffer is preferred, usually in (0.3 M) sucrose or (150 mM) NaCl. In general, sucrose-based buffers are known to enhance protein stability and reduce potential non-native protein aggregation, but due to preferential hydration on the protein surface, electrostatic protein-protein interactions are favored. Conversely, salt-based buffers influence the net charge of charged amino acid side groups on the protein surface, thus having a bias towards more hydrophobic-based interactions^28^.

Co-IP is the most commonly employed biochemical assay to assess protein-protein interactions especially from native tissue. Its main drawback is the requirement for highly specific antibodies validated for use in IP and immunoblotting^10,16,18^. Thus, recombinant proteins are often tagged with a peptide epitope, *e.g*., influenza hemagglutinin (YPYDVPDYA) or human cMyc (EQKLISEEDL), for which high-affinity specific antibodies are commercially available. If desired, the immunoprecipitating antibody can be chemically conjugated onto the Protein-A resin to avoid its elution and detection during the immunoblotting stage that may obscure the co-precipitated protein^16^; to achieve this, we have successfully used the chemical cross-linker 3,3'-dithiobis(sulfosuccinimidylpropionate)^24^. It is imperative that an appropriate detergent is included in the IP buffer and the insoluble material of homogenized cells is removed by centrifugation to minimize non-specific binding (step 3.1.3). The choice and concentration of detergent are important considerations: stronger detergents and/or higher concentrations will substantially reduce non-specific binding but may also abolish X:Y protein interaction, whereas lower concentrations or milder detergents may allow a weak interaction to be observed but may result in excessive non-specific binding. Intermediate strength detergents are preferred, *e.g*., Triton X-100 at 0.5 - 1% concentration. To further reduce non-specific binding, a neutral protein (*e.g.,* bovine serum albumin at 100 µg/ml) can be included in the IP buffer, and/or the cell lysate can be pre-cleared with prior incubation with Protein-A resin alone. The number of washes should be optimized for the X:Y pair tested, typically three 10-min washes with IP buffer (step 3.1.9). In any case, a co-IP sample with non-immune IgG as the immunoprecipitating antibody should always be processed in parallel to serve as negative control (step 3.1.6).

The main advantage of chemical cross-linking is that it informs on the stoichiometric composition of the protein homo-oligomer. Glutaraldehyde is a commonly used cross-linker because it requires no specialized equipment and it generates thermally and chemically stable cross-links between interacting proteins^19,20^. Compounds with free amino groups should be omitted from assay buffers (step 3.2.1) because they will quench the chemical reaction. Glutaraldehyde concentration and reaction time (step 3.2.4) should be optimized for the oligomeric protein complex of interest. The main drawback of this technique, especially when performed on whole cell preparations, is the non-specificity of the chemical reaction that could yield artificial protein aggregates that lack biological significance.

Alternative *in vivo* (*e.g*., fluorescence resonance energy transfer, bi-molecular fluorescence or luminescence complementation) and *in vitro* techniques (*e.g*., size exclusion chromatography, analytical ultracentrifugation, isothermal titration calorimetry) are available for characterization of protein self-association and assessment of oligomerization stoichiometry ^29,30^. Each method has its own advantages and disadvantages, and it may be more suitable for the study of a specific protein depending on protein purification/stability and equipment/reagent availability. The three complementary methods described here in detail, namely Y2H, co-IP and cross-linking, have been used in combination to provide compelling evidence for RyR2 homo-oligomer formation in isolation and within a living cell.

## Disclosures

The authors have nothing to declare.
